# An Audit of Delays in the Management of Non-Metastatic Osteosarcoma at a Tertiary Care Center in South India

**DOI:** 10.7759/cureus.27744

**Published:** 2022-08-07

**Authors:** Gipson Samuel, Aashish Yadav, Prabu Mounisamy, Smita Kayal

**Affiliations:** 1 Department of Orthopaedics, Jawaharlal Institute of Postgraduate Medical Education and Research, Puducherry, IND; 2 Department of Medical Oncology, Regional Cancer Centre, Jawaharlal Institute of Postgraduate Medical Education and Research, Puducherry, IND

**Keywords:** adjuvant chemotherapy, neo-adjuvant chemotherapy, biopsy, mri, waiting, delay, osteosarcoma

## Abstract

Background and objective

Delays in the management of osteosarcoma (OGS) lead to tumor progression and the development of metastasis, resulting in a decrease in overall survival (OS). The primary objective of this study was to determine whether delays occur in implementing the individual steps in the management of OGS in South India.

Methods

In this study, core biopsy reports between October 2019 and October 2021 were retrospectively examined for a diagnosis of OGS. The primary outcome variables in this study were time to MRI, time to biopsy, time to biopsy report, time to neoadjuvant chemotherapy (NACT), time to surgery, and time to adjuvant chemotherapy (ACT). Statistical analysis was performed by comparing the outcome variables with the hypothesized mean.

Results

There were 38 patients with primary non-metastatic OGS. Of these, 92% received NACT, and 74% completed full treatment. The mean time to MRI was 11.3 ± 6.7 days, mean time to NACT was 15.3 ± 12.7 days, mean time to surgery was 31.1 ± 15.3 days, and mean time to ACT was 29.7 ± 10.1 days. Time to MRI was more than seven days in 68% of the cases, while time to NACT was more than seven days in 74%. Time to surgery was more than 21 days in 83% of the cases, and time to ACT was more than 21 days in 82% of the cases.

Conclusion

Based on our findings, there is a significant delay (p<0.05) in time to MRI, time to NACT, time to surgery, and time to ACT. The delay in time to surgery is more than the delay in time to MRI, time to NACT, and time to ACT. The delay is due to a variety of reasons, the most common being the long waiting period at the hospital.

## Introduction

Osteosarcoma (OGS) is the most common primary malignant tumor of the bone in individuals under 30 years of age, with an incidence of five per million persons annually [1]. The five-year overall survival (OS) of patients with OGS is low and stands at 50-60% [2,3]. Hence, there is a constant need to improve OS among these patients. The most important prognostic factors affecting OS are the response to treatment and the development of metastasis [4,5]. Good response to treatment confers a good prognosis, while the development of metastasis confers a bad prognosis. Advancements in chemotherapy regimens and tumor response to treatment have not significantly improved the OS of patients with OGS in the last 30 years [6]. The other important prognostic factor that determines OS is the development of metastasis. Even though the development of metastasis depends more on the intrinsic biology of a tumor, a delay in the management of OGS leads to tumor progression and the development of metastasis, resulting in a substantial decrease in OS [7]. In developing countries, a delay is usually expected due to the poor socioeconomic status of the patients and the long waiting lists in government hospitals [8]. Therefore, any reduction in delay in the management of OGS will improve OS.

Abou Ali et al. have reported that a delay in surgery of more than four weeks after neoadjuvant chemotherapy (NACT) is associated with a significant decrease in event-free survival (EFS) at five years [9], and Yoshida et al. have reported that symptoms lasting for more than four weeks are associated with a significant decrease in OS [10].

The management of OGS involves MRI for local staging, core biopsy for the confirmation of diagnosis, NACT, definitive surgery, and adjuvant chemotherapy (ACT), in that order. In this study, we hypothesize that there is a significant delay at all the individual stages/steps in the management of OGS. The primary objective of this study is to determine whether delays occur in the individual steps in the management of OGS, and the secondary objective is to explore the reasons for the delays. In this study, the timeline of the management of OGS is divided into standard, mutually exclusive individual steps. The division of the timeline will help determine the presence of any delay in individual steps and identify the steps with the maximum delays, which will, thereby, help in designing and implementing possible solutions.

## Materials and methods

In this study, we retrospectively examined core biopsy reports between October 2019 and October 2021 for a diagnosis of OGS. This study was conducted at the Jawaharlal Institute of Postgraduate Medical Education and Research (JIPMER), an institution of national importance under the Ministry of Health and Family Welfare, Government of India. This study was reviewed and approved through a full board review by the Institutional Ethics Committee with the approval number JIP/IEC/2019/408. There are no payments and financial or other relationships to disclose. In this study, dates on which the OGS patients first visited the hospital, underwent MRI, underwent a core biopsy, received the biopsy report, started NACT, completed NACT, underwent definitive surgery, and started ACT were obtained from the patient case files, Hospital Information System (HIS), Picture Archiving and Communication System (PACS), tumor clinic registers, Regional Cancer Centre registers, and orthopedic operation theatre registers. The intervals between the above-obtained dates were calculated using a date calculator (www.timeanddate.com) and entered into a Microsoft Excel workbook (version 2018). Patients whose MRI was not done at our center were excluded from the study. Similarly, patients whose CT scan of the thorax showed lung metastasis were also excluded from the study.

The study subjects were then prospectively contacted over the phone to determine their reasons for delaying treatment. The reasons were then classified into one of the four broad groups of answers: delays due to fear and monetary issues were grouped into personal delay; delays due to waiting for imaging or chemotherapy and the complications of chemotherapy were grouped into medical delay; delays due to waiting for a biopsy or surgery to be conducted and the complications of surgery were grouped into surgical delay; and delays due to the diagnosis of coronavirus disease 2019 (COVID-19) or COVID-19-related travel restrictions were grouped into COVID delay. Patients who had defaulted on treatment at any step were asked to report to the hospital.

**Figure 1:**
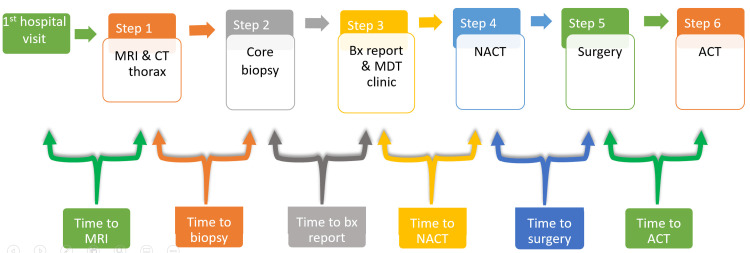
Timeline of the management of OGS showing the operational definitions of the main outcome variables OGS: osteosarcoma; bx: biopsy; MRI: magnetic resonance imaging; CT: computed tomography; MDT: multi-disciplinary tumor; NACT: neoadjuvant chemotherapy; ACT: adjuvant chemotherapy Image credits: Gipson Samuel

The statistical analysis was performed using SPSS Statistics version 26.0 (IBM Corp., Armonk, NY). The primary outcome variables in this study were the duration of symptoms, time to MRI, time to biopsy, time to biopsy report, time to NACT, time to surgery, and time to ACT. The operational definitions of the primary outcome variables are presented in Figure 1.

The primary outcome variables measured were all continuous data, which were analyzed using the Kolmogorov-Smirnov test for normality. Normally distributed continuous data were summarized as mean ± standard deviation (SD). Continuous data that were not normally distributed were summarized as the median and interquartile range (IQR). Categorical data were summarized as proportions. Significant delays in the outcome were determined using the one-sample t-test for normally distributed data and the single-sample Wilcoxon test for skewed data. The acceptable delay at each step was considered as the hypothesized mean for statistical testing. The hypothesized mean time to MRI was considered to be seven days, as imaging of malignant bone tumor is treated as a priority 2 level (urgent) and should be done within one week. The hypothesized mean time to biopsy was considered to be seven days [11]. The hypothesized mean time to biopsy report was considered as 10 days, accounting for the time taken to decalcify bone biopsies. The hypothesized mean time to NACT was considered to be seven days. The hypothesized mean time to surgery was considered to be 21 days, accounting for the time taken for the hematological parameters to become normal. The hypothesized mean time to ACT was considered to be 21 days, considering the time taken for complete wound healing.

For hypothesis testing, the null hypothesis was defined as "no delay in the management of OGS", and a p-value of less than 0.05 was considered statistically significant. To identify delay with a confidence interval of one week and a confidence coefficient of 0.95, the sample size was estimated to be 40. Time to surgery was considered the main outcome variable for sample size calculation, and the population SD was assumed to be 10.5 days, as the expected range was 3-12 weeks. Data associated with COVID-related delays were treated as effect modifiers and were excluded from the analysis.

## Results

Between October 2019 and October 2021, there were 46 core biopsy reports with a diagnosis of OGS. The MRI scans of four patients were not available in the PACS, and the CT scans of the thorax of four other patients showed lung metastasis. These patients were excluded from the study sample. The remaining 38 patients were all cases of primary non-metastatic extremity OGS. In the study sample, 33 out of the 38 patients were between 10 and 35 years of age (87%), 26 out of 38 patients were men (68%), 23 out of 38 patients had OGS on the right side (61%), 30 out of 38 patients had OGS around the knee (79%), and the osteoblastic subtype was the most common histological subtype (56%). Table 1 summarizes the demographic variables of the patients.

**Table 1:** Demographic variables SD: standard deviation

Variables (n=38)		Values
Age, years	Mean ± SD	21.6 ± 14
	Range	6–78
Sex, n (%)	Male	26 (68%)
	Female	12 (32%)
Side, n (%)	Right	23 (61%)
	Left	13 (39%)
Site, n (%)	Distal femur	16 (42%)
	Proximal tibia	14 (37%)
	Proximal humerus	5 (13%)
	Others	3 (8%)
Histological subtype, n (%)	Osteoblastic	21 (56%)
	Chondroblastic	7 (19%)
	Fibroblastic	5 (13%)
	Giant cell rich	2 (5%)
	Small cell	1 (2%)
	Telangiectatic	2 (5%)

**Figure 2:**
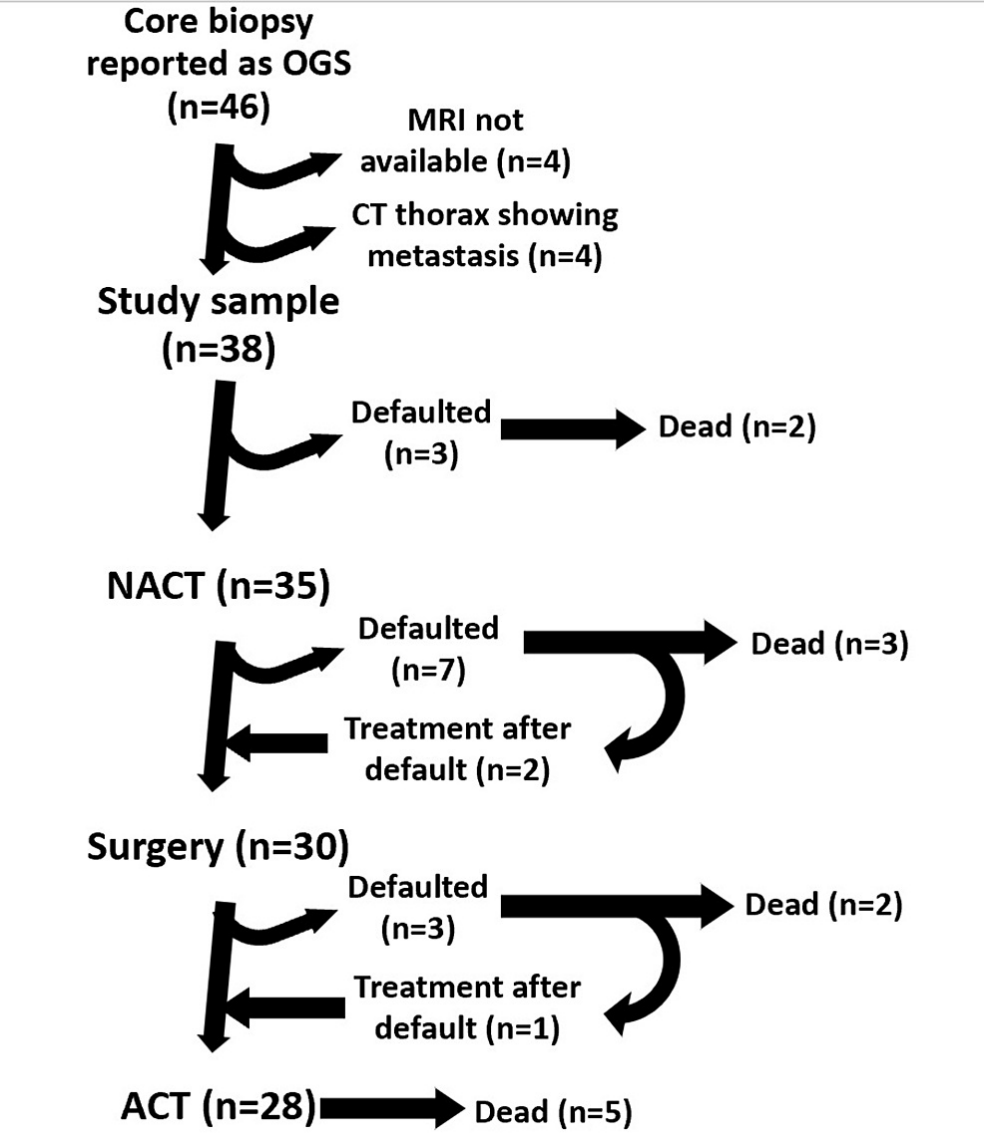
Patient flow diagram OGS: osteosarcoma; NACT: neoadjuvant chemotherapy; ACT: adjuvant chemotherapy Image credits: Gipson Samuel

In the study sample, 35 out of 38 patients received NACT (92%), 30 out of 35 patients underwent surgery (85%), 28 out of 30 patients received ACT (93%), and 28 out of 38 patients completed the full course of treatment (74%). Moreover, 12 out of 38 patients expired during the study period (32%). Figure 2 shows the patient flow diagram.

The mean time to MRI was 11.3 ± 6.7 days. Time to MRI was more than seven days in 26 out of 38 patients (68%) and more than 10 days in 15 out of 38 patients (40%). In most cases, the delay was due to a long waiting list for MRIs (62%). The mean time to biopsy was 8.1 ± 7.7 days. The time to biopsy was more than seven days in 15 out of 38 patients (40%). In most cases, the delay was due to a long surgical waiting list (60%). The mean time to biopsy report was 9.1 ± 4.7 days. The time to biopsy report was more than 10 days in 11 out of 38 patients (29%). In most cases, the delay was due to the long time taken to decalcify the OGS in the pathology laboratory (82%). In two patients, the biopsy report was delayed by 18 days and 27 days due to incomplete biopsy requisition forms. Furthermore, the mean time to NACT was 15.3 ± 12.7 days and the time to NACT was more than seven days in 26 out of 35 patients (74%) and more than 14 days in 13 out of 35 patients (37%). In most cases, the delay was due to a long waiting list (58%). A six-year-old girl and a 19-year-old male defaulted after NACT due to personal reasons. These two patients reported back after 300 days for the continuation of treatment after default. The six-year-old girl defaulted again after surgery. The 19-year-old male developed multiple lung metastases when he reported back for treatment and was managed with palliative care. The mean time to surgery after NACT was 31.1 ± 15.3 days. Time to surgery after NACT was more than 21 days in 25 out of 30 patients (83%) and more than 28 days in 13 out of 30 patients (43%). In most cases, the delay was due to a long surgical waiting list (60%). The mean time to ACT was 29.7 ± 10.1 days. Time to ACT was more than 21 days in 23 out of 28 patients (82%) and more than 28 days in 14 out of 28 patients (50%). In most cases, the delay was due to personal reasons (43%). In one patient, the delay was more than 28 days due to delayed healing of the surgical wound.

The primary outcome variables, except for the duration of symptoms, were normally distributed after excluding the outliers and effect modifiers. The median duration of symptoms was four months with an IQR of four months and a range of 1-24 months. Due to the COVID-19 pandemic, MRI was delayed in two patients, biopsies were delayed in two patients, NACT was delayed in one patient, surgery was delayed in three patients, and ACT was delayed in two patients. The six-year-old girl and the 19-year-old male who defaulted after NACT and reported back after 300 days for surgery were treated as outliers and excluded from the analysis of the time to surgery. A 12-year-old girl defaulted after surgery and reported back for ACT after 100 days. She was excluded from the analysis of the time to ACT. Table 2 summarizes the primary outcome variables.

**Table 2:** Primary outcome variables *A p-value of less than 0.05 is considered significant SD: standard deviation; NACT: neoadjuvant chemotherapy; ACT: adjuvant chemotherapy

S. no	Outcome variable (days)	Summary statistics: mean ± SD	Hypothesized mean (days)	P-value*
1	Time to MRI	11.3 ± 6.7	7	<0.001
			10	0.262
2	Time to biopsy	8.1 ± 7.7	7	0.401
3	Time to biopsy report	9.1 ± 4.7	10	0.236
4	Time to NACT	15.3 ± 12.7	7	<0.001
			14	0.566
5	Time to surgery	31.1 ± 15.3	21	0.003
			28	0.326
6	Time to ACT	29.7 ± 10.1	21	<0.001
			28	0.402

## Discussion

The demographic variables in this study are comparable to other studies from India. The mean age of patients with OGS was 21.6 years (range 6-78 years) in our study. It was comparable to the data published by Rajendranath et al. from the Adyar Cancer Institute, Chennai. In that study sample, comprising 272 patients treated between 1998 and 2008, the median age was 17 years (range 6-52 years) [2]. In 60% of the cases, the OGS presented in the second decade, as in other studies from India [12]. As described in other Indian studies, the patients included in our study were predominantly male [8]. Most cases of OGS (79%) occurred around the knee. This is consistent with the findings from Tata Memorial Hospital, Mumbai, and Adyar Cancer Institute, Chennai, which reported that OGS occurred around the knee in 74% and 89% of cases, respectively [2,12]. The most common histological subtype in this study was the osteoblastic (56%) subtype; this aligns with the findings of a study from Chennai, South India [2]. The median duration of symptoms on presentation was four months (range 1-24 months). A similar delay in presentation has been seen in developed countries such as the Netherlands (median of four months with a range of 1-36 months) [13]. At the All India Institute of Medical Sciences (AIIMS), New Delhi, 42% of patients presented after four months of symptoms [8]. In this study, 29% of OGS cases presented after four months of symptoms. The percentage of patients receiving NACT is comparatively higher in this study, which is likely due to the social improvement in recent years in South India. The percentage of patients receiving NACT in South India is shown in Table 3 [2,3].

**Table 3:** Percentage of patients receiving NACT in South India NACT: neoadjuvant chemotherapy

City, State	Year	Sample size	% of patients receiving NACT
Chennai, Tamil Nadu [2]	1998–2008	272	57.6
Thiruvananthapuram, Kerala [3]	2008–2013	62	88.7
Puducherry	2019–2021	38	92

Time to surgery after NACT was more than four weeks in 13 out of 30 patients (43%) in this study. The same outcome variable in a study from Lebanon showed a delay of more than four weeks in 22 out of the 33 patients surveyed (66.7%) between 2001 and 2012 [9].

In this study, 28 out of 38 patients completed their treatment (74%), and five out of 28 patients (18%) died after the completion of treatment during the study period. The time to ACT in all the above-mentioned five patients had a delay of more than 30 days. In 68%, 74%, 83%, and 82% of patients, there were delays in the time to MRI, time to NACT, time to surgery, and time to ACT respectively. There were delays of more than seven days in the time to MRI and time to NACT due to long waiting periods in the hospital in 62% and 58% of patients, respectively. The time to surgery was delayed by more than 21 days due to long waiting periods in the hospital for 60% of patients. Time to ACT was delayed by more than 21 days in 43% of patients because of personal reasons. A significant delay (p-value of less than 0.05) was observed in time to MRI, time to NACT, time to surgery, and time to ACT. If the hypothesized mean was increased by one week, there was no significant delay in any of the individual steps. This is because the existing delay was not very long and was around seven days for each of the individual steps in the management of OGS. The mean difference in time to MRI, time to NACT, time to surgery, and time to ACT with the hypothesized mean was 4.3, 8.3, 10.1, and 8.7 days, respectively. In other words, the delay in time to surgery was more than the delay in time to MRI, time to NACT, and time to ACT. The sample mean of the primary outcome variables did not show a big difference from the hypothesized mean, but the sample SD was widely dispersed, probably owing to the multifactorial causes of delay in the outcome variables.

A major limitation of this study was the disruption caused by the COVID-19 pandemic. In 2020 and 2021, the COVID-19 pandemic disrupted the functioning of many hospitals and transport services. As a result, COVID-related delay led to many outliers in the data. This was overcome by the exclusion of data associated with COVID-related delays from the analysis. Recall bias could not be eliminated while contacting patients to understand the reasons for the delay. It was minimized by collecting data from the patients and their caregivers; these data were then compared with the data in the patient case files.

The study design was appropriate for answering the hypothesis of this study. Prospective studies performed to analyze delay factors may lead to selection bias. The sample size was adequate to identify a confidence interval of one week. The demographic variables of this study are comparable to other published data from the Indian subcontinent. The primary outcome variables of this study are normally distributed. We believe the findings of this study are relevant for comparison with similar studies in the future.

## Conclusions

Based on our findings, there are significant delays in time to MRI, time to NACT, time to surgery, and time to ACT. These delays are due to a variety of reasons, the most common being the long waiting period. The delay in time to surgery is more than the delay in time to ACT, time to NACT, and time to MRI. Future studies with the aim to determine possible solutions to prevent delay and studies comparing the delay with OS can help prevent such delays and provide insights into the role of delays in the overall prognosis of the patients.
